# Chronic nonbacterial osteomyelitis in children: a multicentre Belgian cohort of 30 children

**DOI:** 10.1186/s12969-022-00698-3

**Published:** 2022-06-13

**Authors:** Sara Kaut, Ine Van den Wyngaert, Davy Christiaens, Carine Wouters, Nathalie Noppe, Nele Herregods, Joke Dehoorne, Lien De Somer

**Affiliations:** 1 Department of Paediatrics, Leuven University Hospitals, Leuven, Belgium; 2 Department of Radiology, Leuven University Hospitals, Leuven, Belgium; 3 Department of Paediatrics, Paediatric Rheumatology and Immune-Inflammatory Diseases, Leuven University Hospitals, Herestraat 49, 3000 Leuven, Belgium; 4 Department of Paediatric Radiology, Ghent University Hospital, Ghent, Belgium; 5 Paediatric Rheumatology, Ghent University Hospital, Ghent, Belgium

**Keywords:** Chronic Non-Bacterial Osteomyelitis (CNO), Chronic recurrent multifocal osteomyelitis (CRMO), Paediatrics, Bone pain, Radiology, Whole-body MRI, Management, Tocilizumab

## Abstract

**Background:**

To evaluate clinical characteristics, imaging findings, therapeutic approach and outcome of paediatric patients with Chronic Non-Bacterial Osteomyelitis (CNO).

**Methods:**

Retrospective review of 30 children diagnosed with CNO at two tertiary care centres in Belgium. Imaging data were evaluated by blinded paediatric radiologists.

**Results:**

Mean age at onset was 10.3 years and mean age at diagnosis was 11.7 years. Bone pain was the leading symptom (29/30 patients). Out of 180 symptomatic lesions, 131 were confirmed on MRI as hyperintense geographic lesions on STIR images at the metaphysis and epiphysis adjacent to growth plates of tubular bones. The most common sites of involvement were the lower limbs, spine, sternoclavicular joint and humerus. For nearly half of the patients (14/30) monotherapy with NSAIDs was sufficient to obtain remission. The remaining 16 patients received second-line therapy: bisphosphonates (*n* = 15/30), disease-modifying antirheumatic drugs (*n* = 7/30), etanercept (*n* = 4/30) and tocilizumab (*n* = 1/30). Remission was reached after a mean time of 37.6 months in 26/30 patients. The prognosis was worse for patients with spinal involvement, resulting in more long-term sequelae.

**Conclusions:**

We present a multicentre paediatric cohort of 30 CNO patients. A typical pattern of bone involvement could be found on MRI. NSAIDs were administered as first-line treatment. Second-line strategies included bisphosphonates, corticosteroids, methotrexate, etanercept and tocilizumab.

**Trial registration:**

Retrospectively registered. Registratienummer EC KUL: MP018023

## Background

Chronic nonbacterial osteomyelitis (CNO), also known as Chronic Recurrent Multifocal Osteomyelitis (CRMO) was first described in 1972 [1]. It is a rare autoinflammatory disorder, characterized by chronic, sterile osteomyelitis in one or more bones presenting with recurrent episodes of bone pain. The metaphysical area of long bones, spine and clavicle are typically involved [2–5]. The pathophysiology still remains unclear. There are arguments that a dysregulation between Toll-like receptor 4 (TLR4), mitogen-activated protein kinase (MAPK) and inflammasome signalling, leads to an imbalance between pro- (IL-1, IL-6, TNF-α, IL-20) and anti-inflammatory (IL-10, IL-19) cytokine expression from monocytes, resulting in bone inflammation and osteolysis [6]. The existence of monogenic autoinflammatory diseases with osteomyelitis as main clinical feature, reports of CNO occurring in twins and siblings and the association of CNO with other inflammatory diseases like psoriasis and inflammatory bowel disease (IBD) in first degree relatives suggest a genetic predisposition [7, 8].

The incidence of CNO is not reliably know, with a wide reported range from less than 1/100.000 children/ year [3] to rates comparable with bacterial osteomyelitis (10–80/100.000 children/year) [9]. It primarily affects children and adolescents but it can persist in adulthood or present later in life. The average age at onset of symptoms is 10 years [2–5]. Systemic symptoms including fever, malaise, weight loss and fatigue can be found in a third of CNO patients. Extra-skeletal manifestations; arthritis, palmoplantar pustulosis, psoriasis vulgaris, IBD, Takayasu arteritis and renal disease are associated in 10 to 30% of patients [3, 4, 8, 10]. CNO can be complicated by vertebral compression fractures, kyphosis and leg length discrepancy when it is not recognized early or treated adequately.

Diagnosis of CNO is challenging and has a broad differential diagnosis including infection, malignancy (primary bone tumours and leukaemia/lymphoma), Langerhans cell histiocytosis, juvenile idiopathic arthritis and metabolic disorders (including hypophosphatasia). Laboratory investigations, imaging studies and histology can be useful for diagnosis and monitoring of the disease [3, 4, 9, 11].

Treatment of CNO patients has been mostly empiric. Nonsteroidal anti-inflammatory drugs (NSAIDs) are often the first-line treatment. Corticosteroids, bisphosphonates, tumour necrosis factor-alpha (TNF-α) blocking agents and methotrexate are reported as second-line treatment with variable success [3, 12–14].

The aim of this study was to evaluate demographic and clinical characteristics, magnetic resonance imaging (MRI) findings and therapeutic features in paediatric patients with CNO at two tertiary centres in Belgium. Radiological data were reviewed in detail to detect specific signs that can distinguish CNO from other diseases such as infection, benign bone lesions and malignancies. We correlated clinical symptomatic lesions with inflammatory signs on imaging.

## Methods

A retrospective review of medical records of 30 paediatric patients diagnosed with CNO between January 1996 and December 2017 at the paediatric rheumatology departments of the University Hospitals of Leuven and Ghent. The study was approved by the Supervisory Committee on Medical ethics of the “Master of medicine (Leuven)” programme and the Ethics Committee UZ Ghent.

The diagnosis of CNO was defined as the presence of unifocal or multifocal bone inflammatory lesions with radiological and/or histopathological characteristics compatible with this diagnosis, having excluded infectious, oncological or other inflammatory diseases. A clinical bone lesion was defined as an area of bone pain reported by the patients.

Clinical characteristics such as age, sex, age at onset of symptoms, personal and family history, treatments with their effectiveness and side effects and sequalae were registered. Remission was defined as the absence of pain and inflammatory signs in blood and/or disappearance of inflammation on imaging for at least six months.

Laboratory evaluations, imaging and histology from bone biopsy were collected. On MRI, lesions were defined as areas of bone marrow edema, which implicates an abnormal hyperintensity on short tau inversion recovery (STIR) images or/and abnormal hypointensity on T1-weighted images and/or areas of contrast enhancement. Lesions were evaluated for location, periosteal reaction and association with joint or/and soft tissue involvement. On radiographic images, lesions were classified according to their density (osteolytic, sclerotic or mixed) with or without periosteal reaction, hyperostosis, cortical expansion or ill-defined margins. Imaging data were compared to clinical findings. All imaging data were reviewed by a radiologist blinded to the clinical data.

## Results

### Demographic data and clinical characteristics

A total of 30 patients were enrolled in the study. 73% (*n* = 22) were female and 27% (*n* = 8) were male, with a gender ratio of 2.8:1. Mean age at onset of symptoms was 10.3 years (range 5.8–16.3) and mean age at diagnosis was 11.7 years (range 5.9–16.9) with a mean diagnostic delay of 17 months (range 0.5–64). Symptoms reported by the patients and finding on physical examination are summarized in Table 1.

**Table 1:** Clinical data

Bone pain	29/30 (97%)
Bone pain worsening at night	24/30 (80%)
Loss of function	22/30 (73%)
Morning stiffness	7/30 (23%)
Fever (> 38,5 °C)	3/30 (10%)
Weight loss	7/30 (23%)
Fatigue	14/30 (47%)
Stop sport activities	15/30 (50%)
Pain provoked by trigger (trauma,viral infection, stress)	8/30 (27%)
Pain on palpation	22/30 (73%)
Swelling of the bone	20/30 (67%)
Restricted range of motion	14/30 (47%)
Associated arthritis	5/30 (17%)

Mean number of clinical lesions per patient was 1.7 (range 1–7) at onset and 6.0 (range 1–19) over the course of the disease. Twenty patients (67%) presented with a unifocal disease at onset, although during follow-up 15 of them evolved to a multifocal disease. Lesions were asymmetric in 24/30 patients (80%). In total, 180 clinical lesions were reported. Most frequently involved were the knee (18%), followed by the spine and pelvis (13%), proximal femur (9%), ankle (7%), clavicle, humerus, distal tibia and sternoclavicular joint (each 5%). Number and distribution of lesions are described in Table 2.

**Table 2:** Number and distribution of inflammatory bone lesions

	CLINICAL		RADIOLOGY	
	(n)	(%)	(n)	(%)
Clavicle	9	5%	13	8%
Shoulder	6	3%	5	3%
Humerus	9	5%	11	6%
Radius	3	2%	0	0%
Ulna	0	0%	0	0%
Elbow	5	3%	2	1%
Wrist	4	2%	0	0%
Hand	0	0%	0	0%
Pelvis	23	13%	28	16%
*Sacroiliac joint*	*12*	*7%*	*13*	*8%*
*Ileum*	*6*	*3%*	*9*	*5%*
*Pubis*	*5*	*3%*	*6*	*3%*
Hip	4	2%	7	4%
Femur	16	9%	22	13%
Knee	33	18%	23	13%
*Distal femur*	*11*	*6%*	*13*	*8%*
*Knee*	*9*	*5%*	*0*	*0%*
*Proximal tibia*	*13*	*7%*	*10*	*6%*
Tibia	9	5%	10	6%
Fibula	4	2%	5	3%
Ankle	12	7%	7	4%
Metatarsals	3	2%	1	1%
Spine	23	13%	23	13%
*Cervical*	*3*	*2%*	*2*	*1%*
*Thoracic*	*10*	*6%*	*10*	*6%*
*Lumbar*	*5*	*3%*	*4*	*2%*
*Sacral*	*5*	*3%*	*7*	*4%*
Scapula	3	2%	1	1%
Ribs	4	2%	3	2%
Sternum	9	5%	10	6%
Mandible	1	1%	1	1%

Half of the patients had extraosseous manifestations: six patients (20%) had aphthous oral ulcers, five patients (17%) had severe acne, four (13%) psoriasis and four (13%) eczema. Two patients (7%) had Crohn’s disease and one patient (3%) developed malign hypertension caused by Takayasu arthritis. One patient (3%) was diagnosed with synovitis, acne, pustulosis, hyperostosis and osteitis (SAPHO) syndrome. This patient presented at adolescent age with severe acne and pustulosis which improved with oral corticosteroid, MTX and TNF-α blockade.

None of the patients had a family history of CNO. However, other CNO-related diseases were found in patients family members, like rheumatic disease (ankylosing spondylitis, rheumatoid arthritis in 12/30 or 40%), psoriasis (in 7/30 or 23%) and IBD (in 5/30 or 17%).

### Laboratory investigations

Inflammation with elevated C-reactive protein (CRP) concentrations (range 0.4–112 mg/L) and erythrocyte sedimentation rate (ESR) (range 12–101 mm/h) was noted in 18 patients (60%) and 26 patients (87%) respectively. Human leukocyte antigen-B27 (HLA-B27) was detected in two of seven tested patients (29%), one patient had associated arthritis and the other involvement of sacroiliac joint. Antinuclear antibodies and rheumatoid factor were found in none of the tested patients (20 and 13 respectively).

Blood cultures were performed in 17/30 patients (57%) and synovial fluid was analysed in 4 (13%). Both tests remained sterile in all patients. A bone biopsy was performed in 16 patients (53%), showing a mononuclear cell infiltration, mainly plasmocytes in most patients, which is a typical finding for severe chronic inflammation. In none of the cases there was a suspicion of malignancy. Bone biopsy was especially performed in older patients, at a time era where MRI was less available.

### Imaging

Conventional X-ray was performed in all patients, whole-body MRI in 18/30 patients (60%) and targeted MRI in 27/30 (90%). 172 radiological lesions were identified during the follow-up with an average of 5.7 lesions per child (range 1–20). In our cohort conventional radiographs showed osteolytic (36%), sclerotic (24%) or mixed (40%) lesions. Periosteal reaction, hyperostosis and ill-defined margins were seen in respectively 22, 14 and 16% of the lesions. In most cases CRMO showed a typical pattern of bone involvement on MRI: multifocal, hyperintense lesions on STIR images at the metaphysis and epiphysis adjacent to growth plates of tubular bones. Symptomatic and asymptomatic lesions can show the same abnormalities on MRI, for example only bone marrow edema, although, we found that lesions with a periosteal reaction or cortical involvement (respectively 3 and 4%) are mostly symptomatic. MRI showed associated synovitis in 10 joints and soft tissue oedema was described eight times. The most common sites were the pelvis (16%), the knee (13%: distal femur 8% and proximal tibia 6%), the spine (13%), proximal femur (13%), clavicle (8%), humerus, tibia and sternum (each 6%). There were no CNO lesions found in the skull, ulna, radius or hand. Number and distribution of lesions are described in Table 2.

### Combining clinics with imaging

**Fig. 1:**
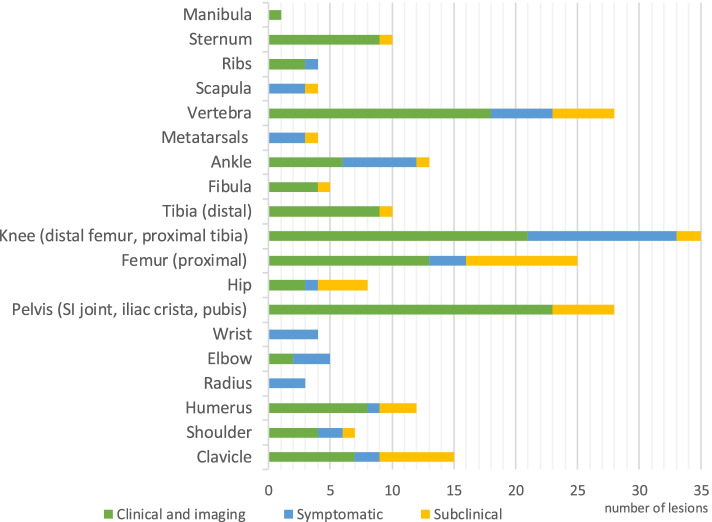
Number of lesions (sub) clinical and on imaging Blue: clinical painful bone lesions (symptomatic). Yellow: subclinical lesions (edema on imaging but clinically asymptomatic). Green: clinical lesions confirmed on imaging

Five patients had a clinical unifocal course of their CNO, in four of them imaging confirmed the unifocal lesion (localizations: clavicle, distal femur, pelvis, sternoclavicular joint). One patient with a clinical unifocal sacral location had an additional, subclinical lesion at the acromion on total body MRI. On the other hand, two patients in our cohort had unifocal involvement on imaging but reported multiple clinically symptomatic sites. Of the 180 clinical painful bone lesions, 131 were confirmed on imaging. For 49 anamnestic painful localizations, no correlation was found on MRI imaging, as they were likely caused by other etiologies such as muscular pain. There were 41 subclinical lesions (edema on imaging but clinically asymptomatic) (Fig. 1).

### Treatment

**Fig. 2:**
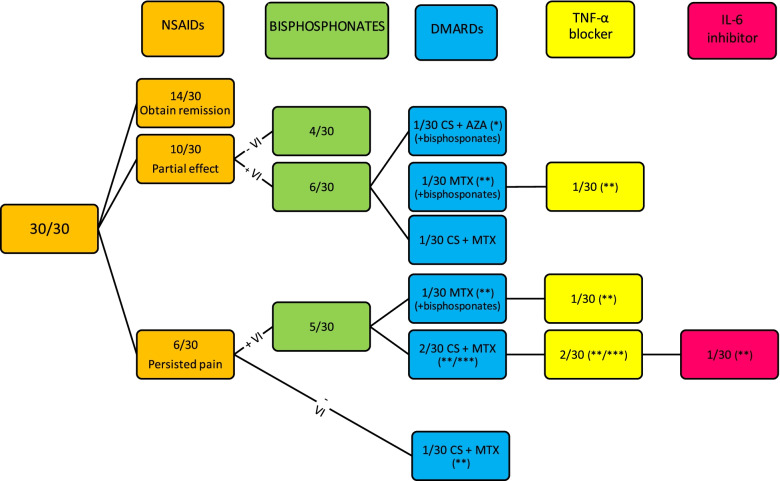
Therapeutic flowchart VI = vertebral involvement, CS = corticosteroids, MTX = methotrexate, AZA = azathioprine. * patient with Crohn’s Disease, ** patient with associated arthritis, *** patient with SAPHO

In terms of treatment all patients received NSAIDs, 15 bisphosphonates (pamidronate), 6 methotrexate, 1 azathioprine, 6 corticosteroids, 4 etanercept and 1 tocilizumab (Fig. 2).

NSAIDs were used as first-line therapy. In 47% of patients, NSAIDs were sufficient to obtain long-lasting remission. Corticosteroids were given due to associated arthritis (*n* = 3), strong inflammatory facial lesions in the patient with SAPHO (*n* = 1), Crohn’s disease (*n* = 1) and as bridging therapy with methotrexate in a patient who developed new lesions under therapy with pamidronate (*n* = 1).

Half of the patients (*n* = 15) received pamidronate (mean of 3.8 cycles, range 1–10 cycles). Eleven of the 16 patients with vertebral involvement, were treated with pamidronate (68%). In three patients, pamidronate was combined with disease modifying antirheumatic drugs (DMARDs) from the start. Nine out of twelve patients (75%) receiving pamidronate without DMARDs, could reduce the NSAIDs during treatment with pamidronate and regain their physical activity.

In our study, seven patients (23%) were treated with a DMARD. One patient with Crohn’s disease was treated with azathioprine, the six remaining patients received methotrexate (10–15 mg/m2/dose once weekly). During treatment with a DMARD, corticosteroids and NSAIDs could be discontinued in three out of seven patients. In the four remaining patients (one with SAPHO, three with associated arthritis), methotrexate was insufficient to control the disease and add-on with a TNF-α blocker (etanercept) was necessary. Etanercept resulted in clinical and biochemical remission in three out of four patients. One patient had ongoing inflammation despite receiving all above mentioned therapies and was finally switched to intravenously tocilizumab in combination with methotrexate with success as long as tocilizumab was given every four weeks.

In 18/30 patients (60%) physical therapy was necessary to improve range of motion and regain normal mobility.

### Outcome

Seven patients (23%) had a vertebral compression fracture. One patient needed spine surgery. Five patients (17%) had a leg length discrepancy. Four patients (13%) needed psychiatric or psychologist support because of emotional stress caused by this disease. The patient with SAPHO experienced more stress because of the extensive cutaneous lesions and was hospitalised in the department of paediatric psychiatry for depression, auto mutilation and suicidal attempts.

Remission was achieved in 26 patients (87%) with a mean time of 37.6 months or 3.1 years (range 7–96 months). Three patients were lost from follow up.

## Discussion

We present a retrospective multicentre case series of paediatric CNO patients and describe their clinical presentation, imaging data, treatment and outcome. To our knowledge this is the first case series from Belgium.

Mean age of onset of disease and diagnosis as well as the ratio of female to male patients and diagnostic delay were similar to data published in previous studies [2–5, 15]. The prolonged delay between onset of symptoms and diagnosis can be attributed to the absence of pathognomonic clinical and laboratory signs in diagnosing CNO, the relatively ignorance of the disease, as well as CNO being a diagnosis of exclusion. Increased attention to the disease has been seen, through which diagnostic delays have been shortened [5]. Our study confirms that local bone pain, worsening at night is the main clinical feature of CNO [2, 3, 8]. Similar to previous observations, local swelling and arthritis were not always present [3, 8]. Systemic symptoms such as fever and weight loss were previously reported in one-third of the patients but were less frequent in our analysis [2–5]. We found no correlation between inflammatory markers and number of lesions/associated arthritis. There was no association between higher inflammatory markers and the use of biological treatments. Some classify CNO in the group of autoinflammatory diseases, others see CNO as part of the spectrum of spondyloarthropathies [4, 14]. HLA-B27 was determined in a small proportion of our cohort, not enabling us to find a correlation with HLA-B27-associated spondylarthritis. CNO can also be associated with other inflammatory diseases such as severe acne, psoriasis and IBD which was confirmed in our patient cohort [3, 4, 8]. None of first- or second-degree relatives of our patients were diagnosed with CNO, which is different from previous reports [3, 4, 7].

As described in previous studies, multifocal pattern of CNO lesions was most common [4] with the clavicle being a typical site for CNO disease [4, 5, 11]. The mean number of painful osseous lesions per patient over the course of the disease was consistent with published data [4, 7, 16, 17]. MRI correlates well with the clinical findings: lesions are most frequently seen in the lower limbs, the spine and pelvis, the clavicle and sternoclavicular joint and proximal humerus as described in other series [3, 4, 7, 18]. The prevalence of vertebral involvement in our study was higher than previously described, although, the reported percentages had a broad range (4–30%) [3, 16, 19].

In our cohort, conventional radiographs showed characteristic lesions of osteolysis, sclerosis or mixed lesions. Radiographic CNO lesions are described in early stages as an osteolytic region and sclerosis mostly occurs in a later stage as part of the healing process, just like the periosteal reaction [3, 11]. But radiography certainly has its limitations; it does not evaluate the entirety of the skeleton and has a very low sensitivity [11, 20].

**Fig. 3:**
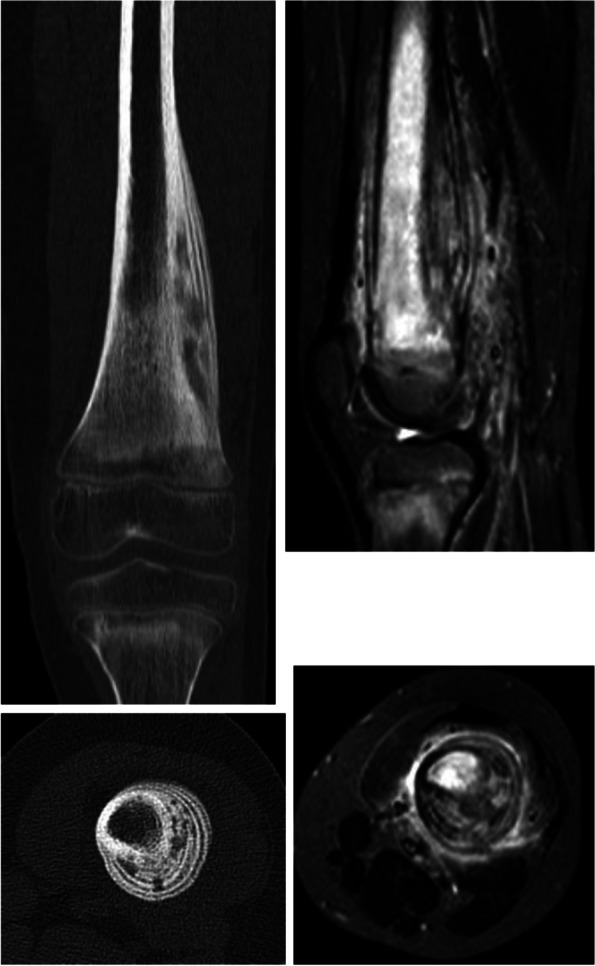
Distal femoral CNO lesion: CT and MRI imaging Left: CT image shows osteolytic region with periosteal reaction. Right: MRI image (STIR) demonstrates bone marrow edema and surrounding soft tissue edema and layers of periosteal reaction

MRI and/or isotopic bone scanning, is the cornerstone for detecting the multifocal pattern, location and distribution of lesions and to exclude the main differentials [9, 20]. Currently, work-up with total body MRI is common and recently consensus-based MRI scoring tool for children with CNO has been developed [21] to improve interrater reliability and agreement of readings. Furthermore, MRI is useful to determine the progression of the disease or the effect of the treatment [18]. Use of total body MRI has improved the sensitivity of detecting CNO lesions and decreased the need for invasive diagnostic procedure like bone biopsy. The threshold for biopsy however must remain low in unifocal lesions and in any case with doubtful diagnosis [5, 9]. There are some MRI sequences that are helpful to differentiate CNO from other diseases. STIR is water sensitive sequence with fat suppression and demonstrates fluid, bone marrow and soft tissue edema, as illustrated in Fig. 3. Bone marrow edema is a typical finding in CNO, in contrast to soft tissues abnormalities which are rare in CNO as seen in our cohort [3, 22]. Another important MRI sequence is diffusion-weighted imaging (DWI). It uses the diffusion of water molecules to generate contrast in MRI images and can be quantitative assessed using the apparent diffusion coefficient (ADC). In some malignant lesions it will result in a low ADC, on the other hand bone marrow edema gives a high ADC [23].

Treatment of CNO is yet to be standardized but there is general agreement that NSAIDS are the best first-line treatment. Uniform guidelines on the second-line treatment for children with CNO are scarce. In 2017 the CNO subgroup of the Childhood Arthritis and Rheumatology Research Alliance (CARRA) published three standardized consensus treatment plans for patients with CNO with insufficient response to NSAIDs and/or the presence of active spinal lesions [24]. Placebo-controlled trials on treatment of CNO are lacking.

In our series, NSAIDs as first-line therapy in all patients, was sufficient to obtain remission in almost half of the patients. High efficacy of NSAIDs was reported by Catalano-Pons et al. where 73% of patients were responders and the effectivity of NSAIDs appeared to be inversely associated with the number of lesions at the onset of the disease [2]. Effectivity of NSAIDs was not correlated with number of lesions in our cohort, but rather correlated with absence of associated arthritis. In a large French study, NSAIDs were also effective in 73% of patients, although effectiveness was defined as an improvement of symptoms, which does not mean the patients were in remission [4]. Other studies had a lower efficacy of NSAIDs [5, 16, 25]. In the different studies definition of efficacy and the described population varies, therefore results are not always comparable. The prospective use of NSAIDs has been evaluated in a single study performed in 37 CNO patients. A favourable clinical course was reported in 43% of patients taking naproxen at 6 months of follow-up; moreover, the total number of clinical detectable lesions was significantly reduced [26].

Bisphosphonates play an important role in the bone remodelling by inhibiting the osteoclast activity; they proved to be anti-inflammatory and reduce pain [12, 27, 28]. In our population, nine out of 12 patients (75%) receiving more than one cycle of pamidronate without associated DMARDs, improved symptomatically. Uniform data on dosage, duration and monitoring of the bisphosphonates in CNO are lacking [27]. Evaluating the effect of bisphosphonates is difficult since different dosing regimes have been used in published studies [12, 27–29].

The most frequently used DMARDs for CNO are methotrexate and sulfasalazine [13, 16, 25]. The reported success rate in inducing remission with methotrexate is variable [5, 24, 25]. In our cohort the use of these conventional DMARDs was rather unsuccessful, four out of the seven patients (57%) only experienced a temporary effect. Standard dose used was similar to that used in juvenile idiopathic arthritis. Low dose methylprednisolone (0.2–0.3 mg/kg) can be used to more rapidly control inflammatory bone pain.

TNF-α blocking agents restore the imbalance between pro- and anti-inflammatory cytokines [13]. Infliximab as well as etanercept and adalimumab are used with variable success [25, 30, 31]. Disease activity seems to be better controlled with TNF-α blocking agents compared to conventional therapy (NSAIDs, corticosteroids and pamidronate) [32]. TNF-α blocking agents also improve acne fulminans and can therefore be recommended as first choice second-line therapy in patients with associated inflammatory skin lesions [33]. In our cohort, four patients (13%) received etanercept in combination with methotrexate, resulting in remission in three patients. Because of the varying results and the lack of guidelines for the use of TNF-α blockers in patients with CNO, we would recommend this therapy in refractory cases.

One patient, with severe inflammation, resistant to all previously described treatments, received intravenous tocilizumab (anti-IL-6 receptor mAb). Tocilizumab, combined with corticosteroids and methotrexate, induced remission after three weeks. To our knowledge, this is the first paediatric patient with CNO who receives IL-6 inhibition. Successful therapy with an IL-6 inhibition is previously reported in adult patients with persistent inflammation under the conventional therapy [34]. IL-6 is known to regulate osteoclast-mediated bone erosion [35].

CNO has generally a good long-term prognosis and resolves often without sequelae [3, 8, 11, 16]. Physical sequelae of CNO described here are in line with other studies where incidence is varying between 20 and 26% [2, 4, 36]. The most common long-term sequelae are vertebral compression fractures with development of spinal misalignments [3, 4, 12, 27]. When the inflammatory lesions are close to the growth plate, CNO can cause a discrepancy in bone length [2, 3, 36]. Early diagnosis and prompt adequate treatment are important in the prevention of long-term sequelae [9]. Psychological stress and depression due to CNO is rarely mentioned in studies, although psychological support seemed to be useful in 1/10 patients in our population. Depression was reported in one patient (2.5%) by Catalano-Pons et al. and Huber showed that CNO affected education in 16% of the patients [2, 36].

Limitations of our study are non-homogeneous retrospective analysis, data collection and patient selection bias (more severe cases) across the two tertiary care centres. The diagnostic modalities and therapeutic options varied between the two centres and response to treatment was based on the interpretation of the treating physician. Due to the retrospective design, missing data were inevitable, particularly for HLA-B27 results. Due to rarity of the disease, the population was rather small, so careful interpretation of the results is necessary.

The results of our case series, facilitated our future diagnosis and treatment of patients with CNO in our centres; i) all patients suspected of CNO will receive a whole body MRI ii) interpretation of whole body MRI by experienced radiologist, using a uniform MRI scoring tool for children with CNO, will decrease the need for bone biopsy iii) NSAIDs remains first line standard treatment, iv) bisphosphonates have proved their role as second line treatment and biologicals are important in refractory cases.

This case series reflects on clinical manifestations, as well as diagnostic investigations, treatment and prognosis of CNO. Additionally we described the first case of a paediatric CNO patient treated with IL-6 inhibition combined with methotrexate.

## Conclusion

Clinical manifestations, laboratory and radiological investigations, treatment and prognosis were described in 30 patients with CNO. Bone pain is the leading symptom in this population. A typical pattern of bone involvement can be found on MRI, through which CNO can be differentiated from other diseases and demonstrates subclinical lesions. NSAIDs are first choice in the treatment of CNO. However, guidelines and consensus about the second-line treatment are still missing. Successful use of IL-6 inhibition was described in one patient resistant to first and second-line treatment. Further studies on treatment strategies and pathogenesis are warranted.

## Data Availability

The datasets generated and/or analysed during the current study are not publicly available (contains patients information).

## Abbreviations

CNO: Chronic Non-Bacterial Osteomyelitis
CRMO: Chronic recurrent multifocal osteomyelitis
MRI: Magnetic resonance imaging
NSAIDs: Nonsteroidal anti-inflammatory drugs
IBD: Inflammatory bowel disease
TNF: Tumour necrosis factor-alpha
IL: Interleukin
STIR: Short tau inversion recovery
CRP: C-reactive protein
ESR: Erythrocyte sedimentation rate
HLA-B27: Human leukocyte antigen B27
DMARDs: Disease modifying antirheumatic drugs
WBC: White blood cells
SAPHO: Synovitis, acne, pustulosis, hyperostosis and osteitis
ADC: Apparent diffusion coefficient
DWI: Diffusion-weighted imaging
CARRA: Childhood Arthritis and Rheumatology Research Alliance
